# The method of exclusion (still) cannot identify specific mechanisms of cultural inheritance

**DOI:** 10.1038/s41598-022-25646-9

**Published:** 2022-12-15

**Authors:** Alberto Acerbi, William Daniel Snyder, Claudio Tennie

**Affiliations:** 1 Division of Psychology, Centre for Culture and Evolution, Brunel University London, Uxbridge, UB8 3PH UK; 2 Faculty of Science, Department of Geosciences, WG for Early Prehistory and Quaternary Ecology, University of Tübingen, Schloß Hohentübingen, Burgsteige 11, 72070 Tübingen, Germany

**Keywords:** Human behaviour, Archaeology, Cultural evolution, Animal behaviour, Anthropology

## Abstract

The method of exclusion identifies patterns of distributions of behaviours and/or artefact forms among different groups, where these patterns are deemed unlikely to arise from purely genetic and/or ecological factors. The presence of such patterns is often used to establish whether a species is cultural or not—i.e. whether a species uses social learning or not. Researchers using or describing this method have often pointed out that the method cannot pinpoint which specific type(s) of social learning resulted in the observed patterns. However, the literature continues to contain such inferences. In a new attempt to warn against these logically unwarranted conclusions, we illustrate this error using a novel approach. We use an individual-based model, focused on wild ape cultural patterns—as these patterns are the best-known cases of animal culture and as they also contain the most frequent usage of the unwarranted inference for specific social learning mechanisms. We built a model that contained agents unable to copy specifics of behavioural or artefact forms beyond their individual reach (which we define as “copying”). We did so, as some of the previous inference claims related to social learning mechanisms revolve around copying defined in this way. The results of our model however show that non-copying social learning can already reproduce the defining—even iconic—features of observed ape cultural patterns detected by the method of exclusion. This shows, using a novel model approach, that copying processes are not necessary to produce the cultural patterns that are sometimes still used in an attempt to identify copying processes. Additionally, our model could fully control for both environmental and genetic factors (impossible in real life) and thus offers a new validity check for the method of exclusion as related to general cultural claims—a check that the method passed. Our model also led to new and additional findings, which we likewise discuss.

## Introduction

To identify a species as cultural, researchers have often resorted to examine the distribution of behavioural and/or artefact forms (henceforth: behaviours) in different groups of the same species, using the so-called method of exclusion: if a behaviour is present in group A, but not (or its frequency is extremely low) in group B, and if this pattern is unlikely to be explained by genetic and/or environmental differences, then this behaviour should be considered as being under some social learning influence. This then means that the species has culture. We call this the *distributional* approach to culture. The distributional approach is at the core of several studies of wild primates, including chimpanzees^1^, orangutans^2^, spider monkeys^3^, gorillas^4^, and bonobos^5,6^, and it has also been used in non-primate species^7^. Research on humans and pre-modern hominins has also adopted, albeit sometimes implicitly, the distributional approach to cultural identification^8,9^. While there now exist additional methods aimed to identify social learning in the wild^10,11^—in this paper we will concentrate on the classic method: the method of exclusion*.*

While it is social learning that leads to culture, different social learning mechanisms lead to different types of culture. Many researchers therefore additionally aim to establish whether a species uses specific social learning mechanisms—as indicative of different types of culture. These researchers have often (though not always, see^12–14^) focused on detecting social learning mechanisms that are able to transmit specifics of (behavioural and/or artefact) forms. Here, the social learning is much more defined—it would have to be social learning that copies specifics of the forms of the behaviour themselves (or of the know-how, underlying the behaviour). Henceforth, we will define social learning mechanisms that copy specifics of forms as copying social learning, or—for brevity—as “copying”.

The focus on copying as defined above and as related to animal culture first appeared near the end of last century. Initially it was even claimed that only those species capable of copying (via specific mechanisms of social learning capable of doing so; e.g., imitation of behavioural forms) should be regarded capable of culture more generally. Species lacking copying abilities were supposed to be regarded as incapable of culture. Instead, such species were claimed to maximally show “traditions” instead—that is, as long as they do apply at least some other “non-copying” types of social learning^15^. While this particular terminology suggestion (i.e. culture vs. tradition) did not catch on, the underlying idea of copying-derived differences remains influential, not least because such copying is often associated with the claim of uniquely developed^16^ and/or evolved^17^ human copying capacities. Perhaps even more importantly, these copying-based approaches often causally link such copying abilities—directly and/or indirectly—to the existence of complex cumulative culture in humans^17–21^. An absence of such copying, some researchers claim, keeps cultural reach principally bounded^22^—even in cases where non-copying social learning further channels individual learning over time^21,23^.

Instead of being based on the observation of naturally-occurring behavior distributions (as is the focus of the distributional method), the copying-based approach often relies on controlled experimentation, for example experiments on social learning of novel tool use ^22^ and novel behavioural patterns e.g. ^24^ by apes and on prehistoric stone tool production by modern humans^25–27^.

These (and other) approaches all have merits, not least because they can highlight different aspects of culture(s). Problems arise, however, when a conclusion deriving from one approach is used to validate the other. In particular, we focus here on issues concerning the inference of the necessary existence of copying (as defined above) from observations of distributions of behaviours (i.e. from outcomes of the distributional approach). Note that we are not the first to caution against this inference^28–31^. However, previous accounts used verbal arguments that we here supplement, replicate and test via an individual-based model. It therefore seemed useful to create a model-based replication of the verbal arguments against the copying-inference-from-distributions (henceforth: copying inference). Additionally, we reasoned that a modelling approach would be especially beneficial due to its own reproducibility, its clarity (as the agents’ properties will be fully knowable) and for the possibility to use the model outputs to recreate by-now-iconic outcomes of the method of exclusion applied to the question of ape culture^1^. Therefore, we chose to (re-)illustrate the invalidity of the copying inference, using the specific example of non-human great ape (henceforth: ape) culture.

There are two further, more specialised, reasons for our ape target choice. First, there are good quantitative data on the distribution of behaviours among wild ape populations that can be used to inform our model. In particular, we guide our model development through chimpanzee culture^1^, but a similar logic, and similar data, exists in other primate studies^2–5,32^. Second, the existence of ape culture under the distributional approach is uncontroversial, but the debate on their ability to faithfully copy specifics of forms (i.e. copying) still continues; especially when such specifics are beyond the innovative power of individuals (see below).

Apes clearly are able to socially learn—connectedly, they also clearly have cultures—but it remains an open question what kind(s) of culture(s) they have, and in particular whether their cultures frequently or ever are based on (or even require) copying, as we define it^23,32,33^. Experiments have abundantly shown that humans are capable of copying the specifics of even arbitrary behavioural and artefact forms—importantly, *even of forms they could not produce on their own*—indeed humans routinely copy in this way cross-culturally^34,35^. In contrast, the evidence for such copying by apes remains highly debated—in particular when it comes to copying of forms that no individual ape could produce in the absence of models—i.e. forms for which it is logically necessary to copy the underlying specifics^22,24,36^. Indeed, the evidence for copying in apes has weakened in recent years. While apes can be trained, intentionally in so-called Do-As-I-Do studies^37^, or unintentionally, via human enculturation^38^, to copy form specifics (i.e., copying), their spontaneous abilities of copying in absence of such human training (which is necessarily the case in wild apes) has so far proven elusive in adequately controlled experiments. Namely, when other learning mechanisms were made impossible and when—crucially—the tasks required subjects to copy forms that they were not already able to realistically produce on their own (i.e. the clearest example of copying possible) apes reliably fail to copy these “novel forms"^22,24,36^. Untrained, unenculturated apes not only fail to copy novel action forms, but they also fail to copy physical forms that they could not produce on their own^22^—though more studies on physical form copying beyond the individual reach would be needed.

The debate around ape copying is therefore not a debate on whether apes are incapable of some variants of social learning (nor is it a debate around whether some non-ape species copy). Clearly, even unenculturated, untrained apes are very capable social learners with respect to other—i.e. “non-copying” social learning mechanisms. For example, these apes are avid know-where (via local enhancement), and know-what (via stimulus enhancement) social learners. Instead, the continuing debate revolves around whether apes are ever spontaneously engaged in copying specifics of behavioural or artefact forms—and in particular, whether they copy forms that they could not have produced on their own (forms that are not in their latent repertoire) even when they are motivated to do so (i.e. in culturally unconnected populations, such as in appropriately powered baselines with sufficient levels of motivation)^20,23^.

This leaves social learning relating to behavioural or artefact forms that *remain* in the realistic reach of individuals in the absence of know-how models. There is an ongoing debate around whether such social learning should likewise count as—and be labelled as—copying. This notion remains controversial. Some claim that this type of learning *should* *not* be called copying^21,23,29^, some claim that it *should* e.g.^39^—yet others use different terminology altogether; to however likewise mark this very difference^40^. In our view, Sperber^29^ raised a valid point when he claimed it is best not to call the social release of a form that could have been *individually* derived copying. Sperber suggests the term “triggering” for such learning. Indeed, also to us it would seem prudent that, unless specifics of forms are “causally copied” —in that the observing individual would not have derived at the same or similar form in the absence of this copying—the social learning type should not be called copying ^20^. Taking inspiration from Sperber’s example of laughter contagion and following the alternative logic to the end, one might otherwise have to count yawning contagion as copying, too—an awkward conclusion that we wish to avoid. Moreover, to the extent that one accepts this difference in terminology—between triggering and copying—apes show rather clear evidence for the former, but not the latter^23^ (see also above). Here, we therefore count cases of triggering not as copying, but subsume them under the general label of non-copying social learning.

In this context and debate, any type of data supporting the case of ape copying (as defined here) therefore becomes especially relevant. This is where our focus on the “copying inference” comes into play. The remaining ape copying inferences (i.e. conclusions for ape copying, mainly based on outcomes of the distributional approach) are now some of the *last remaining* claims that (supposedly) support the idea that apes can and do spontaneously use copying. Yet, given that the copying inference has long known to be invalid, it becomes necessary to critique remaining instances of this inference—but also to help guard against this inference being made in future. All this in an effort to prevent this important debate—around ape copying—to be skewed by inadmissible claims.

As mentioned, the copying inference has already been criticised in the past. This critique was even mirrored in the main ape studies that used the distributional approach—these studies cautioned in various ways and depths against this kind of illogical inference e.g.^1,4^. It may thus be thought that no further critique of the copying inference is necessary. This would be incorrect, however. First, not all publications about ape culture caution against this inference—creating a risk that readers themselves derive at the copying inference from the presented data ^41^. Second, despite the relatively frequent calls for caution, the unwarranted copying inference continues to be made—explicitly—in the literature. For example, the literature contains the copying-inference for observed population differences in chimpanzee (and hominin) tool use generally^42^, as well as for more specific cases, such as chimpanzee termite fishing^32,43,44^ and chimpanzee ant-dipping^45^, chimpanzee object-in-ear behaviour^46^ and certain plant processing behaviours in gorillas^47^. The copying inference is also strongly hinted at in a recent paper on chimpanzee underground honey extraction^48^. Explicit copying inferences have likewise appeared in another field—in the field of archaeology, drawing on population differences in stone core rotation patterns in the Oldowan^8^. Various copying inferences were likewise used by one of the reviewers to this manuscript during the review process. Lastly, we also frequently encounter the copying inference in verbal debates (e.g. after talks). In short, despite frequent calls for caution, the problem persists—the copying inference continues to be made. This calls for additional correctional approaches, of which our current manuscript is one such attempt.

We therefore set out to model the distributional approach under conditions of strict absence of copying. However, the individual agents in our model needed to be equipped with some skills and abilities - including social learning -  which we matched to those spontaneous skills and abilities that are clearly present in apes. In our model, we considered an hypothetical ape species, “oranzees”. The skills and abilities (including learning abilities) present and absent in oranzees are therefore a direct reflection of the empirical evidence regarding real apes. First, we equipped agents with individual learning skills. Untrained, unenculturated apes in captivity proved individually able to develop a range of behavioural forms shown by wild apes—all in the entire absence of any form demonstrations (i.e. in the absence of copying opportunities). In this way, such apes showed spontaneous individual reinnovations of a variety of wild forms (including tool forms), such as, e.g. stick pounding^49^, nutcracking^50^, and algae scooping^51^. Such reinnovation of key know-how aspects of these behaviours is not restricted to captive apes. A recent meta-review—using a different method from the method of exclusion (i.e. the method of local restriction) —concluded that wild apes likewise were able to re-innovate behavioural forms across culturally unconnected populations (which could therefore not have copied each other)^52^. Consequently, our oranzees likewise were given the ability to individually innovate (and re-innovate) behavioural forms.

Next, we equipped oranzees with non-copying social learning. Captive apes^53^ and wild apes^54^ increase their individual *likelihood of expression* of specific latent forms upon contact with others expressing these forms, often indirectly via the social transmission of related (e.g., know-what, know-where) information— “socially mediated reinnovation”^49^, and also due to triggering^21,23^ - sensu^29^. Therefore, we likewise built in increased likelihoods of oranzees’ individual expression of specific latent forms upon social encounters of them with others who already express these forms—i.e. we built in non-copying social learning. That is, oranzees, like wild and captive apes, can be socially led to individually develop similar forms/know-how—as long as they stem from their latent repertoires (in a process more resembling catalysation or crystalisation than copying).

As discussed above, there is no evidence that apes spontaneously copy behavioural and/or artefact forms that they could not have principally have individually innovated, if sufficiently motivated. Thus, in our model, oranzees do not copy innovations that they could not have innovated on their own. For example, they cannot copy novel forms as can derive from past cultural evolution (there is no such evolution in our model). Instead, oranzees (presumably like wild and captive apes^21–23^) instead draw from a latent form repertoire—individually and socially—from a limited repertoire range^52^ from which each form can principally develop on an individual level. Likewise, our oranzees were made unable to copy specific details of behaviours—indeed, such details were not even specified in our model.

Our model is therefore designed with apes—and ape skills; but also known ape limitations—in mind. It was designed to test whether distributions-based inferences of ape copying are valid. In addition, our model might provide valuable insights into the cultures of some other animal species (in particular those who might be unable to copy as defined here), as well as to some pre-modern hominin cultures as inferred from the archaeological record^27^. In particular, demonstrating via our model that cultural distributions in apes are unable to pinpoint the specific copying of forms beyond individual reach would mean that similar distributions in pre-modern hominins also cannot pinpoint hominin copying of such forms^55,56^. By extension, the model we present here could be applicable as a predictive hypothesis for modeling the site-level distribution of forms seen from the Paleolithic record^8^ as population-level distributions are harder to detect in non-living species.

What follows is our oranzee model. We are particularly interested in determining whether oranzee skills and abilities alone—i.e. excluding copying, as defined here—would lead, over time, to patterns that would be described as cultural by the distributional approach to culture. If they do, this would replicate earlier verbal models criticising inferences for underlying copying based on such patterns.

## Model

We build an individual-based model that reproduces a world inhabited by six populations of “oranzees”, a hypothetical ape species. The model is spatially explicit: the oranzees populations are located at relative positions analogous to the six chimpanzees sites in Whiten et al.^1^ This is important to determine the potential genetic predispositions^57,58^ and ecological availabilities associated with their possible behavioural or artefact forms, or “behaviours” (see below for more details). Population sizes are also taken from the sites in Whiten et al.^1^ Following Lind and Lindenfors^59^, we use data from Wrangham et al.^60^, and we define population sizes as $N = \left\{ {20;42;49;76;50;95} \right\}$.

Oranzees are subject to a simplified age-dependent birth/death process, broadly inspired by descriptions in Hill et al.^61^ A time step $t$ of the simulation represents a month in oranzees’ life. Starting when they are 25 years old ($t = 300$), there is a 1% probability an oranzee will die each month (maximum lifetime is capped at 60 years, i.e., $t = 720$). The number of individuals in the population is fixed, so each time an oranzee dies it is replaced by a newborn.

A newborn oranzee does not yet show any behaviour, but is individually capable of developing them. Behaviours can be developed at each time step, among the 64 possible behaviours in our model (see below). The process of development of behaviours is influenced by: (i) the oranzee's ‘state’, which depends on the behaviours an individual already expresses, (ii) the frequency of the behaviours already expressed in the population (non-copying social learning, e.g. via “socially mediated reinnovation” and “triggering”), and (iii) the genetic propensity and ecological availability locally associated with the behaviour. At the beginning of the simulations, the populations are randomly initialized with individuals between 0 and 25 years old.

### Oranzees’ behaviours and state

In the oranzees’ world, 64 behavioural form are latently possible (loosely modelled on the 65 behaviours coded in Whiten et al.^1^, but making it an even number for modelling convenience). Behaviours are divided into two categories: 32 social and 32 food-related behaviours. These figures were chosen to resemble the behavioural categories considered in Whiten et al.^1^ Behaviours serve oranzees to fulfil various biological functions. Oranzees have a ‘state’ that is based on how many functions are fulfilled in the two main categories of social and food-related behaviours.

In the case of social behaviours, we further assume four sub-categories (‘play’, ‘display’, ‘groom’, ‘courtship’; note that these labels are only meant to be evocative), each with eight possible different behaviours that serve the same function. A function is considered fulfilled if an oranzee shows at least one behaviour out of the eight in the sub-category. Oranzees have a ‘state’ that is based on how many of the four functions are fulfilled. An oranzee has a state value of $0.25$ if, for example, it shows at least one behaviour in the category ‘play’, and none of the others, and a state value of $1$ if it shows at least one behaviour in each sub-category. $p_{{{\text{social}}}}$, the probability to innovate a social behaviour, is drawn from a normal distribution with mean equal to $1 - state_{{{\text{social}}}}$. The idea is, roughly, that the fewer social functions are fulfilled, the higher the probability to innovate a social behaviour, and vice-versa. Notice the probability of innovating a social behaviour is thus independent from the specific sub-category.

Food-related behaviours are analogously divided into sub-categories. Differently from social behaviours, there is a variable number of behaviours in each sub-category. In addition, sub-categories are associated to two different ‘nutrients’, *Y* and *Z*. Here, individuals need to balance their nutritional intake, so that their optimal diet consists in a roughly equal number of food for one and the other nutrient. The state, for food-related behaviours, depends on the total amount of food ingested *and* on the balance between nutrients. The state is calculated as the sum of each sub-category fulfilled (as above, for this to happen there needs to be at least one behaviour present) minus the difference between the number of sub-categories providing nutrient *Y* and the number of sub-categories providing nutrient *Z*. We normalize the state between $0$ and $1$, and, as above, $p_{{{\text{food}}}}$ is then calculated as $1 - state_{{{\text{food}}}}$.

### Non-copying social learning

At each time step, all oranzees have a probability—calculated as described above—for individual acquisition of both social and food-related behavioural forms. But *which* behaviour? The specific behavioural form an oranzee will acquire depends both on the frequency of the behaviours that are already present in the population, and on the ecological availability and genetic propensity associated to the behavioural form (see below). A further parameter of the model, $S$, controls the probability that each aquisition is socially mediated/triggered. When an aquisition is socially mediated/triggered, the probability of innovating each behaviour $B_{i}$ is weighted by its proportional instances in the population among all behaviours of the same category (the 32 social behaviours or the 32 food-related behaviours). That is, the frequency of behavioural forms can socially “catalyse” more individual innovations of the same behaviour: common behaviours are more likely to be individually *re*innovated based on this social learning. When the innovation is not socially mediated/triggered, the probability of innovating each behaviour is random. In all cases, only one behaviour per individual can be innovated at each time step.

### Genetic propensity and ecological availability

Finally, the behaviour chosen through the procedure described above is actually innovated or not according to its genetic propensity and, in the case of food-related behaviours, ecological availability.

Genetic propensity is a probability $p_{g} \left( {0,1} \right)$, assigned independently for each of the 64 behaviours (and not to be confused with instincts). A parameter of the model, $\alpha_{g}$, determines the probability that the genetic propensity of each behaviour is equal for all the six populations or whether is different. If the probability is equal, $p_{g}$ is randomly drawn from a uniform distribution between 0 and 1. If it is different, we assign the propensity using a geographical gradient. We choose a random point and calculate its distance to each population. Distances are then transformed to $p_{g}$ by rescaling them between 0 and 1, so that for the farthest site where $p_{g} = 0$, the associated behaviour cannot possibly be expressed (see SI). Notice that $\alpha_{g} = 0$ does not mean that there are no genetic influences on the behaviour, but that there are no *differences* between the populations with regard to this aspect.

Ecological availability is, similarly, a probability $p_{e} \left( {0,1} \right)$ that represents the likelihood of finding a resource, or its nutritional value, in each site. Ecological availability is assigned only to food-related behaviours, and it is calculated in the same way of $p_{g}$, using the parameter $\alpha_{e}$ to determine the probability of ecological availability being different or equal in the six populations.

### Model’s output

We run simulations for $t_{{{\text{max}}}} = 6000$ (corresponding to 500 years of oranzee-time, and sufficient to reach equilibrium in the traits distribution). For each simulation, following Whiten et al.^1^, we classify each behaviour, in each population, as:

- *Customary*: a behaviour observed in over 50% of individuals in at least one age class (see SI for how age classes are defined in our model).
- *Habitual*: a behaviour observed in at least two individuals across the population.
- *Present*: a behaviour observed in at least one individual across the population.
- *Absent*: a behaviour not observed even once in the population.
- *Ecological explanations*: a behaviour that is absent due to a complete lack of local ecological availability (i.e., in our model, associated to $p_{e} = 0$).

Notice that one category in Whiten et al.^1^ (*unknown*, i.e., “the behaviour has not been recorded, but this may be due to inadequacy of relevant observational opportunities”) does not apply in our case, because we have complete knowledge of the output of the simulations.

Finally, to test how well our model compares to wild apes, we calculate the same “patterns” described in Whiten et al.^1^:

- *A*: behaviour absent at no site (i.e., present at every site).
- *B*: behaviour not achieving habitual frequencies at any site.
- *C*: behaviour for which any absence can be explained by local ecological factors.
- *D*: behaviour customary or habitual at some sites yet absent at others, with no ecological explanation, i.e., behaviours defined as “cultural”.

Further details of the model implementation and of how outputs are processed are available in SI. The full code of the model allowing to reproduce all our results, plus a detailed description of the model development is available in a dedicated GitHub repository, at https://github.com/albertoacerbi/oranzees.

## Results

We studied the parameter conditions of moderate to high environmental variability (i.e., $\alpha_{e}$ from 0.5 to 1) and zero to moderate genetic differences (i.e., $\alpha_{g}$ from 0 to 0.5). We ran 20 simulations for each combination (for a total of 600 runs). For all, reinnovation is socially mediated/triggered ($S = 1$). The results show that various combinations of parameters reliably produce behaviours considered “cultural” (pattern D) according to the distributional approach, despite a complete absence of form copying mechanisms. The exact number of cultural behaviours detected by the distributional approach depends on parameter settings.

**Figure 1:**
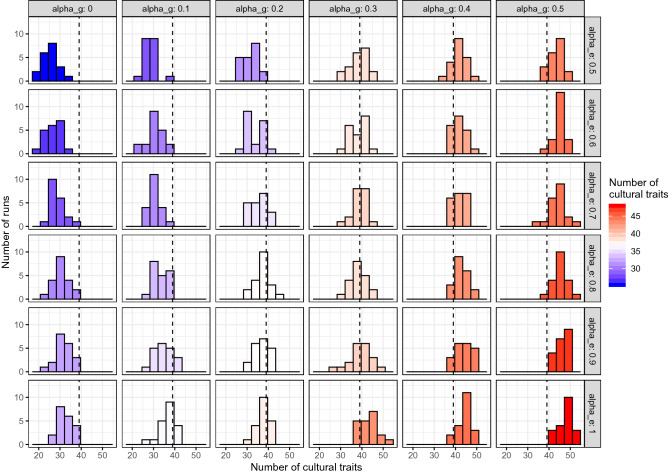
Number of cultural traits in oranzees, when varying ecological and genetic diversity. Red color indicates simulation runs that produced more than 38 cultural traits (the number of cultural traits identified in 1); blue color indicates simulation runs that produced less than 38 cultural traits. For all simulations, $S = 1$, $\alpha_{e}$ and $\alpha_{g}$ as indicated in the plot. $N = 20$ runs for each parameters combination. See SI for other values of $S$, $\alpha_{e}$, and $\alpha_{g}$, including all equal to zero.

**Figure 2:**
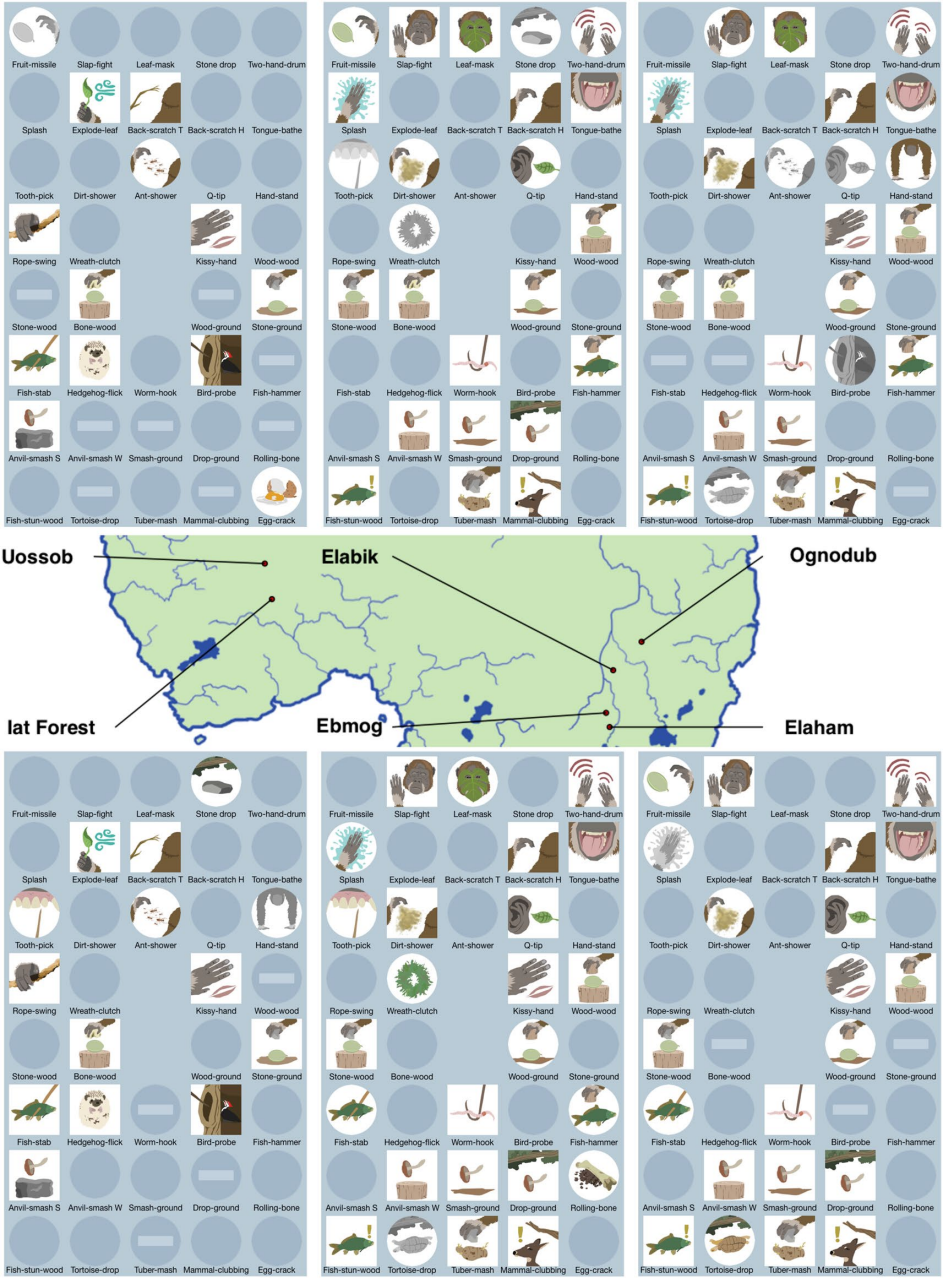
Example of a simulation run that produces 38 cultural traits ($S = 1$, $\alpha_e = 0.8$, and $\alpha_g = 0.2$). Color icons indicate customary behaviours; circular icons, habitual; monochrome icons, present; clear, absent; horizontal bar, absent with ecological explanation. The names of the behaviours are only evocative, see SI for a complete list. (The map was created using the software R (https://www.r-project.org ) and with artwork from WDS.)

Whiten et al.^1^ contained a now-classical—even iconic, at least among ape culture researchers—graphical depiction of the main outcomes of their distributional approach to chimpanzee culture. This particular figure type is able to depict, at one glance, not only the number of cultural behaviours, but also their relative distribution across populations, together with their classification (e.g. habitual vs. customary). We therefore likewise use 38 cultural behaviours as a benchmark to illustrate our results (see Fig. 1). In Fig. 2, we similarly visualize the output of a run of our model in which 38 cultural behaviours were found, including how exactly they were classified in each of the six simulated populations.

We also analysed the effect of the parameter $S$ (proportion of socially mediated/triggered reinnovations), in three conditions (see Fig. S4): (a) no genetic differences and intermediate ecological differences (compare to the high-left corner of Fig. 1, where with $S = 1$ simulations produce less than 38 cultural behaviours), (b) one of the conditions that produce 38 behaviours, namely $\alpha_{e} = 0.8$ and $\alpha_{g} = 0.2$, and (c) intermediate genetic differences and high ecological differences (compare to the low-right corner of Fig. 1, where with $S = 1$ simulations produce more than 38 cultural behaviours). As expected, decreasing *S* decreases the number of cultural behaviours. Conditions where, with $S = 1$, there were more than 38 cultural behaviours could still produce the same amount of cultural behaviours, given that not all reinnovations are socially mediated/triggered.

Finally, we ran 100 simulations for one of the conditions where the output can produce 38 cultural behaviours ($\alpha_{e} = 0.8;\alpha_{g} = 0.2,S = 1$). In each simulation, we recorded, for each population, the number of behaviours (habitual + customary + present) that are also classified as cultural (see Fig. S6). We find a small, but significant, correlation between population size and number of cultural traits ($p < 0.001,\rho = 0.2,N = 600$). In other words, our model reproduces an effect of cultural *ac* cumulation (i.e., increased number of expressed behaviours^62^) relative to population size possibly found in real populations^59^. This effect, too, is therefore found in the absence of any copying.

## Discussion

We developed an individual-based approach to examine—with a model—whether observable distributions of cultural traits can be produced in the absence of social learning mechanisms that transmit specifics of behavioural and/or artefact forms even beyond individuals’ reach (“copying”). Replicating and supporting verbal arguments made in the past, our model shows that population-level distributions of cultural traits, while supporting conclusions for the existence of some type(s) of culture, are not necessarily a product of copying. This finding illustrates once more that copying inferences derived from such distributions alone are invalid.

Though specific values of the outputs, such as the total number of cultural traits, depend on the details of the implementation choices (e.g. repertoire size), our model helps to investigate how the general pattern—copying being unnecessary to produce distributional patterns indicative of culture(s) of some type(s) —interacts with different factors. To illustrate our point, we focused on the distribution reported in Whiten et al.^1^, a representative study of primate culture. We modelled some details of the original wild ape study, including demographic and spatial features, as well as effects of genetic propensity and ecological availability on the behaviours of oranzees. Given the widespread existence of non-copying variants of social learning across the animal kingdom, and given relevant data from previous ape studies, we also included socially mediated reinnovation and triggering, where in both cases social learning merely catalyses individual reinnovation of forms, without specifics of forms being copied—and without copying being strictly necessary to produce target forms—of behaviours and/or artefacts^21,23,29,51^.

Notice that the way in which we implemented individual—but socially mediated/triggered—reinnovation is not equivalent to “unbiased copying”. In our model, reinnovation is implemented by sampling among a number of forms that can be said to be *already present* (at least *latently*) in the individual potential repertoire. With $S > 0$, the sampling is biased by the frequency of forms already expressed in the population, realising a type of (non-copying) social influence. In a model of cultural transmission of the specifics of forms—in a copying model—one would instead have a naïve individual (in our analogy, with an empty repertoire) *copy* forms from other individuals, typically by copying the specifics of the forms that these others show. Importantly, this would even be logically necessary whenever these forms would be outside of the copying subject’s potential and realistic individually reachable repertoire^20^. The fact that the effects of socially mediated/trigggered individual reinnovations alone—i.e. in our model—seem indistinguishable (in terms of population-level outputs) from unbiased cultural transmission is indeed one of our main points. We believe that some of the possible confusion between the two processes may derive from the fact that in standard cultural evolution models social learning is often black-boxed, i.e., as long as the process produces the expected population-level distribution of traits, the details of the process at the individual-level are often considered unimportant. This may be true in the short term, and the short term only—as cultural evolution may hinge on (even derive from) such specifics.

The results of our model show, first, how genetic propensity and ecological availability impact the formation of cultural patterns. Intuitively, the lower the genetic and ecological diversities, the lower the number of traits that are classified as “cultural” according to the distributional method. Notice, however, that even in the entire absence of any ecological and genetic variation, i.e., with $\alpha_{e} = 0$ and $\alpha_{g} = 0$, some cultural traits occur, according to the distributional method (see Fig. S7). Given that this type of exclusion is impossible in real life, this outcome of our model somewhat reinforces the validity of the method of exclusion as a method to detect culture more generally (i.e. in the absence of further inferences regarding underlying learning mechanisms).

Second, we focused on the effects of mechanisms of socially mediated reinnovation/triggering, that is, we assumed that members of our hypothetical species, oranzees, had a probability to individually (re-)innovate a form—stochastically linked to how many other oranzees in their own population were already expressing this form. This makes ecological sense. Mere correlated aspects of these forms (e.g. presence of sticks near prey) can act as indirect cues that can lead to *individual* reinnovation of associated forms in others. For example, a know-*what*-to-eat (e.g. that termite mounds contain eatable *termites*) could drive the individual learning of the know-*how*-to-access the termites (at least, as long as individuals are also motivated to eat termites). Triggering, too, can lead to more instances of these forms in others—without specifics having been copied^23,29^. While these are realistic assumptions^63^, our results demonstrate that not even these socially mediated/triggered effects are strictly necessary to produce distributional patterns that are usually interpreted as cultural. Namely, given certain combinations of parameters, such as higher genetic and ecological diversities, analogous population-level patterns can likewise be obtained even when oranzees are not at all socially influenced in any way by other individuals in their populations (see Fig. S4). That is, patterns that the method of exclusion regards as cultural can also come about when not even non-copying social learning is at work^11^. This is a finding of our model that somewhat *lowers* the validity of the method of exclusion. However, in real life, in most cases, socially mediated reinnovation/triggering *will *likely affect apes—not least, given that they are clearly capable of such learning—and such effects are likely still required to best explain the observed differences in form frequencies between ape populations in real life, where each such population (to a degree) shares genes and environment^41^.

Incidentally, our model more than just allows the determination of whether ape culture patterns derived from the method of exclusion (the distributional method) can exist in the absence of copying (as defined here). It also allows testing of whether other patterns can come about—patterns that are the focus of another, more restricted method that likewise examines distributions of forms within a focus on culture. Namely, the recently published method of local restriction^52^ aims to detect cases not only where behavioural forms are patchily distributed (as in the method of exclusion), but also where the distribution is localized—i.e. *locally* restricted (e.g. behaviour *X* only appearing in one single population of a single species). When this method was applied to the case of ape cultures, it was found that the vast majority of behavioural forms that were previously claimed to be locally restricted in some way were not actually locally restricted. Instead, these ape forms were found to repeat elsewhere, in culturally unconnected populations (often, but not always, of the same species). These repetitions were also found in wild apes. All this is supportive of the hypothesis that these types of forms, or know-how, are part of the apes’ latent behavioural repertoire^21–23,52^. However, in applying this new method to apes, empirically a few cases remained which appear, currently at least, locally restricted in apes. Due to various reasons (e.g. a lack of clear evidence for copying in apes; see introduction), these few remaining cases were regarded as likely false positive outcomes of the method of local restriction by the original authors^52^.

Finding locally restricted forms in our model—even once—would principally support this particular conclusion made by the original authors of this method; namely that *rare *positive cases of locally restricted behaviour need not be based on copying. This support would follow, given that copying was entirely absent from our model. Indeed, rare cases exist of locally restricted behaviours in our model. For example, in the model run depicted in Fig. 2, there are two cases where a behaviour only ever occurs in one single oranzee population (oranzee behaviours number 55 and 64: “egg crack” and “rolling bone”). That is, there can indeed be rare cases of locally restricted behaviours even in the absence of copying. This therefore supports the claim of the original authors^52^ that the detection of a low number of locally restricted behaviours is still fully compatible with a lack of copying (at least, unless other data supports this notion independently).

Finally, our model reproduces a reported correlation between population size and number of cultural traits in the six populations^59,64,65^. Notice that this correlation too was necessarily brought about without any copying, so it is likewise advisable to refrain from inferring underlying learning mechanisms^66^ to explain such patterns. These patterns may simply be due to the fact that socially mediated/triggered reinnovation also produces random drift in segregated groups, confirming the similarity of population-level patterns produced by unbiased copying^67^. Note, however, that the magnitude of the effect we found is small, as the differences in size between populations was also modest.

It is important to be explicit on our main result. Our model does not, and cannot, show that copying mechanisms are *not* causing some or even all of the cultural distributions observed in wild apes. Tests of this specific claim requires different methods (which are however all pointing away from underlying copying; see introduction) also see^52^. Our main finding is instead—replicating verbal claims made before us—that such patterns *do not necessitate* copying mechanisms. Therefore these patterns (still) cannot be used to infer copying—other, non-copying social learning mechanisms, proved sufficient to generate such distributions in our model. We hope that we have further illustrated—here with the help of a model—that the observation of such patterns (the distributional approach) cannot by itself suffice for infering the existence of form copying mechanisms beyond the individual reach. In summary, the copying inference remains invalid—despite its continuing use in the literature (see introduction).

Given the increasing evidence suggesting that apes do not have cultures based on copying (see introduction), it remains unclear as to when and how these copying mechanisms originated. Indeed, the problem of inferring social learning mechanisms from distributions also applies to archaeology, especially in the case of distributions of behavioral traces such as in Early Stone Tools^8,9,68–70^. The method of exclusion alone has clear merits, but with regards to the more specific question of the existence (and timing) of copying, the method does not suffice; other methods (such as the method of local restriction) are needed if we are to better elucidate the learning types behind ape cultures or the cultures of foregone hominin taxa^9,27,52^.

Our results also support and extend previous experimental and modelling work, showing that non-copying mechanisms such as triggering, stimulus enhancement (know-what), and local enhancement (know-where) etc. can create cultural patterns that look much like cultures created by social learning mechanisms that are considered able to copy forms—including novel forms—such as, among others, imitation^71^ and emulation^72^ (note that these - and other - mechanisms can principally transmit such forms, but to do this they need to be present also to a level with sufficient power to actually do so). More generally, our model once again suggests general caution when deriving individual-level mechanisms from population-level patterns. Cultural systems, as many others, exhibit equifinality: the same global state can be produced by different local underlying processes^73–75^. The observation of patterns of cultural traits that differ in different populations (distributional approach) can not be directly used to infer the underlying mechanisms. Other methods, models and experiments remain crucial to test the plausibility of inferences going from global to local properties.

## Supplementary Information

Supplementary Information.

## Data Availability

All codes for running the simulations and reproducing outputs are available at https://github.com/albertoacerbi/oranzees.

## References

[1] Whiten A (1999). Cultures in chimpanzees. Nature.

[2] van Schaik CP (2003). Orangutan cultures and the evolution of material culture. Science.

[3] Santorelli CJ (2011). Traditions in spider monkeys are biased towards the social domain. PLoS ONE.

[4] Robbins MM (2016). Behavioral variation in gorillas: Evidence of potential cultural traits. PLoS ONE.

[5] Samuni L, Wegdell F, Surbeck M (2020). Behavioural diversity of bonobo prey preference as a potential cultural trait. eLife.

[6] Hohmann G, Fruth B (2003). Culture in bonobos? Between-species and within-species variation in behavior. Curr. Anthropol..

[7] Rendell L, Whitehead H (2001). Culture in whales and dolphins. Behav. Brain Sci..

[8] Stout D, Rogers MJ, Jaeggi AV, Semaw S (2019). Archaeology and the origins of human cumulative culture: A case study from the earliest Oldowan at Gona, Ethiopia. Curr. Anthropol..

[9] de la Torre I (2019). Searching for the emergence of stone tool making in eastern Africa. Proc. Natl. Acad. Sci..

[10] Kendal RL, Kendal JR, Hoppitt W, Laland KN (2009). Identifying social learning in animal populations: A new ‘option-bias’ method. PLoS ONE.

[11] Franz M, Nunn CL (2009). Network-based diffusion analysis: A new method for detecting social learning. Proc. Biol. Sci..

[12] Canteloup C, Cera MB, Barrett BJ, van de Waal E (2021). Processing of novel food reveals payoff and rank-biased social learning in a wild primate. Sci. Rep..

[13] Neadle D, Allritz M, Tennie C (2017). Food cleaning in gorillas: Social learning is a possibility but not a necessity. PLoS ONE.

[14] Schuppli C, van Schaik CP (2019). Animal cultures: How we’ve only seen the tip of the iceberg. Evol. Hum. Sci..

[15] Galef BG (1992). The question of animal culture. Hum. Nat..

[16] Heyes C (2018). Cognitive Gadgets.

[17] Tomasello M (1999). The Cultural Origins of Human Cognition.

[18] Henrich J (2015). The Secret of Our Success: How Culture Is Driving Human Evolution, Domesticating Our Species, and Making Us Smarter.

[19] Boyd R (2017). A Different Kind of Animal: How Culture Transformed Our Species.

[20] Buskell, A. & Tennie, C. Mere Recurrence and Cumulative Culture at the Margins. *Br. J. Philos. Sci. *10.1086/717776?journalCode=bjps (in press).

[21] Tennie C, Bandini E, van Schaik CP, Hopper LM (2020). The zone of latent solutions and its relevance to understanding ape cultures. Biol. Philos..

[22] Tennie C, Call J, Tomasello M (2009). Ratcheting up the ratchet: On the evolution of cumulative culture. Philos. Trans. R. Soc. B.

[23] Tennie, C., Hopper, L. M. & Schaik, C. P. van. On the origin of cumulative culture: Consideration of the role of copying in culture-dependent traits and a reappraisal of the zone of latent solutions hypothesis. In *Chimpanzees in Context* 428–453 (University of Chicago Press, 2021). 10.7208/9780226728032-022.

[24] Clay Z, Tennie C (2018). Is overimitation a uniquely human phenomenon? Insights from human children as compared to Bonobos. Child Dev..

[25] Putt SS, Woods AD, Franciscus RG (2014). The role of verbal interaction during experimental bifacial stone tool manufacture. Lithic Technol..

[26] Morgan TJH (2015). Experimental evidence for the co-evolution of hominin tool-making teaching and language. Nat. Commun..

[27] Snyder WD, Reeves JS, Tennie C (2022). Early knapping techniques do not necessitate cultural transmission. Sci. Adv..

[28] Tooby, J. & Cosmides, L. The psychological foundations of culture. In *The Adapted Mind: Evolutionary Psychology and the Generation of Culture* 19–136 (Oxford University Press, 1992).

[29] Sperber, D. *An Objection to the Memetic Approach to Culture* In: Aunger R (ed) Darwinizing culture: the status of memetics as a science. Oxford University Press, Oxford, pp 163–173 (Oxford University Press, 2000).

[30] Morin O (2015). How Traditions Live and Die.

[31] Charbonneau M (2019). Understanding cultural fidelity. Br. J. Philos. Sci..

[32] Boesch C (2020). Chimpanzee ethnography reveals unexpected cultural diversity. Nat. Hum. Behav..

[33] Sterelny, K. & Hiscock, P. Cumulative culture, archaeology, and the zone of latent solutions. *Curr. Anthropol.* **in press**,.

[34] Nielsen M, Tomaselli K (2010). Overimitation in Kalahari bushman children and the origins of human cultural cognition. Psychol. Sci..

[35] Berl REW, Hewlett BS (2015). Cultural variation in the use of overimitation by the Aka and Ngandu of the Congo Basin. PLoS ONE.

[36] Tennie C, Call J, Tomasello M (2012). Untrained Chimpanzees (*Pan troglodytes schweinfurthii*) Fail to Imitate Novel Actions. PLoS ONE.

[37] Hayes KJ, Hayes C (1951). Imitation in a home-raised chimpanzee. J. Comp. Physiol. Psychol..

[38] Buttelmann D, Carpenter M, Call J, Tomasello M (2007). Enculturated chimpanzees imitate rationally. Dev. Sci..

[39] Whiten A, Horner V, Litchfield CA, Marshall-Pescini S (2004). How do apes ape?. Anim. Learn. Behav..

[40] Byrne, R. W. Imitation of novel complex actions: What does the evidence from animals mean? In *Advances in the Study of Behavior* vol. 31 77–105 (Academic Press, 2002).

[41] Kühl HS (2019). Human impact erodes chimpanzee behavioral diversity. Science.

[42] Whiten A, Schick K, Toth N (2009). The evolution and cultural transmission of percussive technology: Integrating evidence from palaeoanthropology and primatology. J. Hum. Evol..

[43] Sanz C, Call J, Morgan D (2009). Design complexity in termite-fishing tools of chimpanzees (*Pan troglodytes*). Biol. Lett..

[44] McGrew WC (2004). Primatology: Advanced ape technology. Curr. Biol..

[45] Whiten A (2000). Primate culture and social learning. Cogn. Sci..

[46] van Leeuwen EJC, Cronin KA, Haun DBM (2014). A group-specific arbitrary tradition in chimpanzees (*Pan troglodytes*). Anim. Cogn..

[47] Byrne RW, Hobaiter C, Klailova M (2011). Local traditions in gorilla manual skill: Evidence for observational learning of behavioral organization. Anim. Cogn..

[48] Estienne V, Robira B, Mundry R, Deschner T, Boesch C (2019). Acquisition of a complex extractive technique by the immature chimpanzees of Loango National Park, Gabon. Anim. Behav..

[49] Bandini E, Tennie C (2019). Individual acquisition of “stick pounding” behavior by naïve chimpanzees. Am. J. Primatol..

[50] Bandini E, Grossmann J, Funk M, Serrano AA, Tennie C (2020). Naïve orangutans (*Pongo abelii* & *Pongo pygmaeus*) individually acquire nut-cracking using hammer tools. Am. J. Primatol..

[51] Bandini E, Tennie C (2017). Spontaneous reoccurrence of “scooping”, a wild tool-use behaviour, in naïve chimpanzees. PeerJ.

[52] Motes-Rodrigo A, Tennie C (2021). The Method of Local Restriction: In search of potential great ape culture-dependent forms. Biol. Rev..

[53] Huffman MA, Hirata S (2004). An experimental study of leaf swallowing in captive chimpanzees: Insights into the origin of a self-medicative behavior and the role of social learning. Primates.

[54] Hobaiter C, Poisot T, Zuberbühler K, Hoppitt W, Gruber T (2014). Social network analysis shows direct evidence for social transmission of tool use in wild chimpanzees. PLoS Biol..

[55] Tennie C, Braun DR, Premo LS, McPherron SP (2016). The Island Test for Cumulative Culture in the Paleolithic.

[56] Tennie C, Premo LS, Braun DR, McPherron SP (2017). Early stone tools and cultural transmission: Resetting the null hypothesis. Curr. Anthropol..

[57] Forss SIF, Willems E, Call J, van Schaik CP (2016). Cognitive differences between orang-utan species: A test of the cultural intelligence hypothesis. Sci. Rep..

[58] Gumert MD (2019). Prevalence of tool behaviour is associated with pelage phenotype in intraspecific hybrid long-tailed macaques (*Macaca*
*fascicularis*
*aurea* × *M*. *f*. *fascicularis*). Behaviour.

[59] Lind J, Lindenfors P (2010). The number of cultural traits is correlated with female group size but not with male group size in chimpanzee communities. PLoS ONE.

[60] Wrangham, R. W. Why are male chimpanzees more gregarious than mothers? A scramble competition hypothesis. In *Primate Males: Causes and Consequences of Variation in Group Composition* 248–258 (Cambridge University Press, 2000).

[61] Hill K (2001). Mortality rates among wild chimpanzees. J. Hum. Evol..

[62] Dean LG, Vale GL, Laland KN, Flynn E, Kendal RL (2014). Human cumulative culture: A comparative perspective. Biol. Rev..

[63] Whiten A, van Schaik CP (2007). The evolution of animal ‘cultures’ and social intelligence. Philos. Trans. R. Soc. B Biol. Sci..

[64] Tennie C, Call J, Tomasello M (2010). Evidence for emulation in chimpanzees in social settings using the floating peanut task. PLoS ONE.

[65] Langergraber KE (2011). Genetic and ‘cultural’ similarity in wild chimpanzees. Proc. R. Soc. B Biol. Sci..

[66] Henrich J (2004). Demography and cultural evolution: How adaptive cultural processes can produce maladaptive losses: The Tasmanian case. Am. Antiq..

[67] Cavalli-Sforza LL, Feldman MW (1981). Cultural Transmission and Evolution.

[68] Shipton C, Nielsen M, Di Paolo LD, Di Vincenzo F, De Petrillo F (2018). The acquisition of biface knapping skill in the Acheulean. Evolution of Primate Social Cognition.

[69] Shipton C, Groucutt HS (2020). The unity of Acheulean culture. Culture History and Convergent Evolution: Can We Detect Populations in Prehistory?.

[70] Shipton C, White M (2020). Handaxe types, colonization waves, and social norms in the British Acheulean. J. Archaeol. Sci. Rep..

[71] Matthews LJ, Paukner A, Suomi SJ (2010). Can traditions emerge from the interaction of stimulus enhancement and reinforcement learning? An experimental model. Am. Anthropol..

[72] Reindl E, Apperly IA, Beck SR, Tennie C (2017). Young children copy cumulative technological design in the absence of action information. Sci. Rep..

[73] Acerbi A, Van Leeuwen EJ, Haun DB, Tennie C (2016). Conformity cannot be identified based on population-level signatures. Sci. Rep..

[74] Barrett BJ (2019). Equifinality in empirical studies of cultural transmission. Behav. Processes.

[75] Perreault C (2019). The Quality of the Archaeological Record.

